# Discal cysts and pseudocysts: Single center experience

**DOI:** 10.1016/j.inpm.2023.100278

**Published:** 2023-09-09

**Authors:** Vineet V. Gorolay, Brandon K.K. Fields, Vinil N. Shah

**Affiliations:** a Department of Radiology and Biomedical Imaging, University of California, San Francisco, USA

**Keywords:** Discal cyst, Pseudocyst, Back pain, Radiculopathy, Image-guided intervention

## Abstract

**Introduction:**

Current knowledge of intervertebral discal cysts is restricted to case reports and surgical case series, typically in young adult males presenting with back pain and radiculopathy.

**Objective:**

We review our single-center experience to describe presentation, management, and outcomes of these rare lesions.

**Methods:**

We performed a retrospective electronic search of our institution database using key words “discal cyst,” “disc cyst” and variations. Clinical presentation, imaging findings, management and outcomes were reviewed and tabulated.

**Results:**

Nine patients were identified (4 female), with mean age 49.1 years. Three patients had prior surgery at the level of the cyst. Seven patients presented with back pain, five with additional radiculopathy, one patient with radiculopathy alone, and one asymptomatic. Most discal cysts occurred at L5-S1, were left-sided, paracentral in location with a T2 hypointense rim and variable enhancement. One patient underwent primary cyst resection. Amongst 6 patients who underwent primary image-guided procedures, two had sustained pain relief, three proceeded to cystectomy, microdiscectomy and/or posterior decompressive surgery, and two were lost to follow-up.

**Conclusion:**

Our retrospective cohort includes a wider age group with more heterogeneous clinical features, treatment approaches and response to therapy than that described in the literature. CT or fluoroscopy-guided steroid injection provided short-term symptomatic relief with several cases managed definitively with surgery. Further research is required to better understand and manage these rare lesions.

**Clinical impact:**

Discal cysts and pseudocysts occur in a wider range of patients and with more heterogenous presentations than previously described. Imaging-guided intervention can provide short-term symptomatic relief, but further research is required to optimize long-term management.

## 1 Introduction

Discal cysts are rare extradural cysts which communicate with the intervertebral disc [1]. These have been most frequently described in young adult males presenting with back pain and radiculopathy. They are distinct from disc extrusions, which primarily contain extruded nucleus pulposis; facet synovial cysts, which communicate with facet joints in the setting of facet arthrosis; and perineural sleeve cysts, which are CSF-filled dilations of the nerve root sheath. Current knowledge of discal cysts is restricted to case reports and retrospective surgical case series [[2], [3], [4], [5]]. We review our single-center experience to describe presentation, management, and outcomes of these rare lesions.

## 2 Materials and methods

A retrospective electronic search of our institution radiology database from inception until February 2023 was performed using mPower Clinical Analytics (Nuance Communications, Inc; Burlington, MA). Key words included “disc cyst,” “discal cyst,” “pseudocyst” and variations. The study was approved by our Institutional Review Board (IRB) and compliant with the Health Insurance Portability and Accountability Act (HIPAA), with informed consent waived. No funding was received. Two authors (VG and BF) then reviewed reports to identify candidate cases with discal cysts or pseudocysts, excluding reported mimics such as Tarlov cysts, perineural cysts or synovial cysts. Electronic medical records were reviewed using APeX (Advanced Patient-Centered Excellence, Epic Systems; Verona, WI) to collect clinical data including age, gender, prior surgery, symptoms, and examination findings at time of presentation and follow-up. Procedure and operation reports, pain scores (visual analogue scale range between 0 and 10), follow up duration and outcomes were also reviewed. Imaging was reviewed using Visage 7 (Visage Imaging, Inc; San Diego, CA) to collect data including imaging modality, intervertebral disc level, side, location, lesion dimensions, volume, morphology. In cases confirmed with magnetic resonance imaging (MRI), signal characteristics on T1-weighted (T1W), T2-weighted (T2W) sequences and gadolinium enhancement were collated. In cases confirmed with CT, mean attenuation was measured in Hounsfield units. The clinical and imaging findings were reviewed independently by two trainee radiologists (BF, VG) with one and six years of neuroimaging experience respectively, and the results were de-identified and tabulated. Any discrepancies were resolved by discussion with the senior author.

## 3 Results

Our institutional database search encompassed 10 years (2013–2023) during which period 58,332 patients underwent MRI lumbar spine and 58,869 patients underwent MRI cervical spine. Nine patients were identified of whom four were female, with age ranging between 30 and 80 years (mean 49.1 years; Table 1). Three patients had a history of prior surgery including microdiscectomy (n = 2) or discectomy (n = 1) for symptomatic lumbar disc herniation at the level of the cyst. Seven patients presented with back pain, of whom five also described radiating leg pain. One patient presented with radiculopathy alone. One patient presented with neurogenic claudication and one with bilateral leg pain in a clear dermatomal distribution. Amongst the six patients with radiculopathy, four reported paresthesia or hypoesthesia and two exhibited decreased sensation to light touch on clinical examination. No patients exhibited bladder or bowel dysfunction. Initial pain scores ranged between 5 and 9 on a visual analogue scale out of 10. In one patient, the discal cyst was identified incidentally on an abdominal MRI for an unrelated indication, and definitively characterized on dedicated lumbar spine MRI.

**Table 1:** Clinical characteristics, initial and definitive treatment of patients with discal cysts. M = male; F = female; PE = paresthesia; HE = hypoesthesia; DTR = deep tendon reflexes; NA = not applicable; NR = not reported; SNRB = selective nerve root block; TFESI = transforaminal epidural steroid injection; ILESI = interlaminar epidural steroid injection; 1° = primary. Full treatment details are available in Supplementary Tables 1 and 2

Case #	Age Sex	Prior Surgery	Symptom Duration	Dermatome	Pain (/10)	PE	HE	Strength (/5)	DTR (Patella/Achilles)	DTR Babinski	Initial Treatment	Definitive Treatment
1	30F	L4-L5 Micro-discectomy	4 weeks	Left L5	9	Y	N	5	+	↓	CT-guided TFESI, cyst aspiration, fenestration	Revision microdiscectomy
2	40F	–	6 months	Left L5	6	Y	N	4	++	↓	Hemilaminectomy, facetectomy, foraminotomy, cystectomy	1° surgery
3	30M	L5-S1 micro-discectomy	6 weeks	Left L5	4	Y	N	NR	++	↓	CT-guided TFESI, cyst aspiration + fenestration	1° CT-guided TFESI, aspiration
4	37F	–	NR	Left S1	NR	Y	Y	5	NR	NR	Fluoroscopy-guided SNRB	1° fluoroscopy- guided SNRB
5	68M	–	4 years	Right > Left	5	Y	Y	5	–	↓	CT-guided SNRB, ILESI	Bilateral laminotomy, foraminotomy, medial facetectomy
6	80F	–	–	–	0	N	N	NR	NR	NR	Conservative	Conservative
7	52F	–	1 week	Left L3	6	N	Y	5	++	↓	Conservative	Conservative
8	63M	L4-L5 discectomy	2 years	NR	NR	NR	NR	NR	NR	NR	Fluoroscopy-guided discography + aspiration	1° fluoroscopy-guided aspiration
9	42M	–	8 months	Right S1	6	N	N	5	+	↓	CT-guided SNRB	Repeat CT-guided SNRB, ILESI

Discal cysts were identified in eight patients on MRI; and in one patient on CT with fluoroscopic and CT discography confirmation (Table 2). MRI was performed at 1.5 T for 6 patients (GE Signa HDxt, Siemens Magnetom Vision or Philips Achieva) and 3.0 T in 2 patients (GE Discovery MR750 and Siemens Magnetom Verio) with all cases including at least sagittal and axial T1W sequences, axial T2W sequences and sagittal fat-suppressed T2 or short-tau inversion recovery (STIR) sequences. Post-gadolinium T1W sequences with fat suppression were obtained in 6 patients.

**Table 2:** Imaging characteristics of discal cysts on MRI or CT. M = male; F = female; Hypo = hypointense signal; Hyper = hyperintense signal; HU = Hounsfield units.

Case #	Age Sex	Level	Side	Location	Max Dimension (mm)	Volume (mL)	T1W	T2W	T1+C	Communicating Channel	Other Features
1	30F	L4-L5	Left	Paracentral	9	2.4	Iso	Hyper	Rim enhancement	Yes	–
2	40M	L4-L5	Left	Paracentral	18	13.6	Iso	Hyper	Rim enhancement	No	Layering T2 hypointense contents
3	30M	L5-S1	Left	Paracentral	12	4.8	Hypo	Hyper	None	Yes	–
4	37F	L5-S1	Left	Paracentral	10	2.3	Iso	Hyper	NA	No	–
5	68M	L3-L4	Left	Paracentral	11	3.2	Iso + Hypo	Hypo + Hyper	NA	No	Fluid-fluid level
6	80F	L4-L5	Left	Anterior	15	5.1	Hypo	Hyper	None	No	–
7	52F	L5-S1	Right	Paracentral	18	5.8	Hypo	Hyper	None	No	–
8	63M	L5-S1	Left	Anterior	16	13.1	–	–	–	Yes (fluoro)	21 HU on unenhanced CT
9	42M	L5-S1	Right	Paracentral	11	3.2	Hypo	Hyper	Rim enhancement	Yes	–

**Fig. 1:**
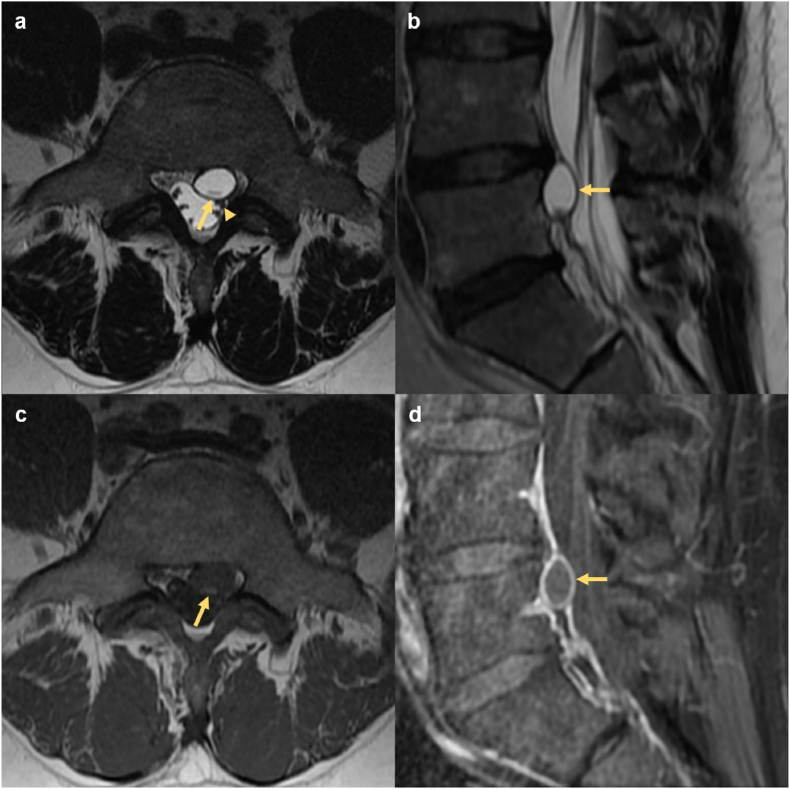
MRI in a 40-year-old male with 6 months of left-sided L5 radicular pain. (a) Axial T2W sequence demonstrates a left paracentral discal cyst (arrow) effacing the left lateral recess and displacing the descending left L5 nerve root dorsally (arrowhead). The cyst is clearly separate from the adjacent left facet joint. (b) Parasagittal T2W sequence demonstrates central T2 hyperintensity within the lesion and a T2 hypointense rim (arrow). (c) Axial T1W demonstrates that the cyst is predominantly T1 hypointense with dependent T1 isointense contents (arrow). (d) Parasagittal post-gadolinium T1W with fat suppression confirms smooth enhancement of the cyst wall (arrow).

**Fig. 2:**
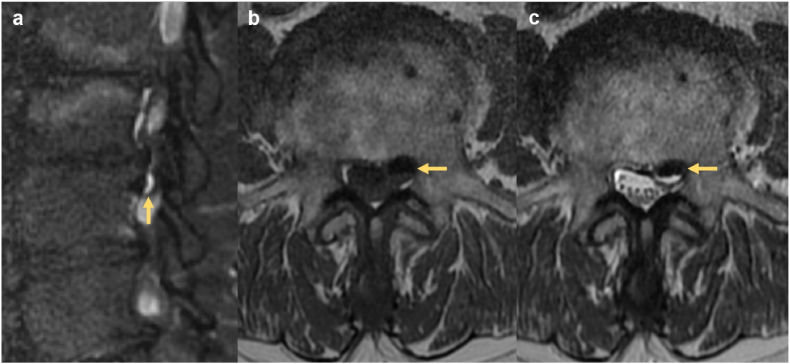
MRI in a 68-year-old male with 4 years of bilateral neurogenic claudication. (a) Parasagittal T2W sequence with fat suppression demonstrates an extradural cystic lesion immediately below the L3-4 disc (arrow). (b) Axial T1W confirms the discal cyst to be intraspinal, extradural, and clearly separate from the adjacent left facet. There is a fluid-fluid level (arrow) consisting of dependent isointense and antidependent hypointense signal components. (c) Axial T2W demonstrates a fluid-fluid level (arrow) with dependent T2 hyperintense signal and antidependent hypointense signal. Severe spinal canal stenosis was present at other levels, not shown.

**Fig. 3:**
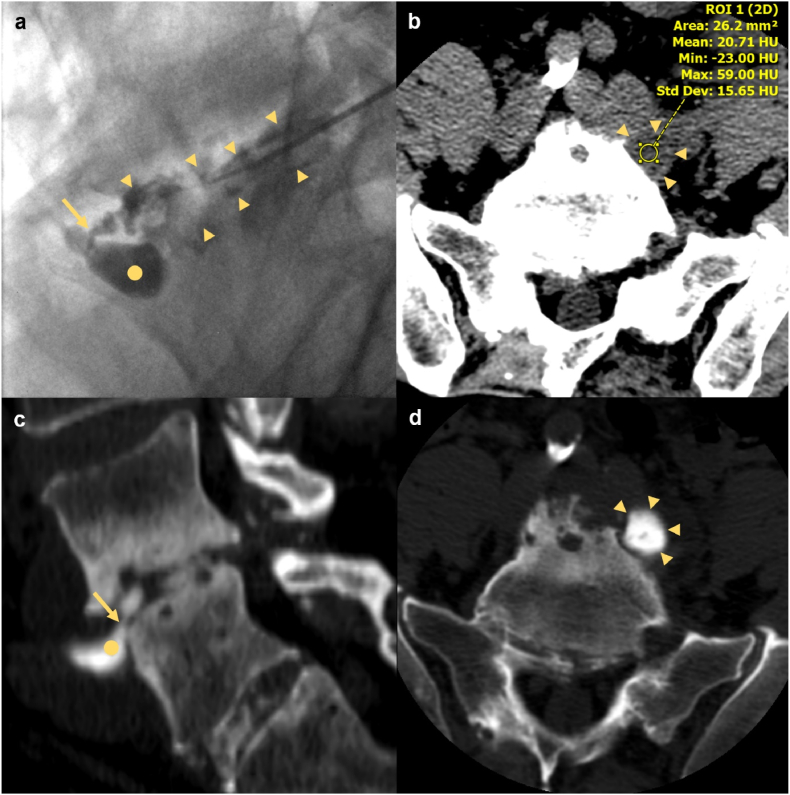
CT and fluoroscopic-guided discography in a 63 year old male with prior L4-L5 discectomy who presented with 2 years of back and bilateral leg pain. (a) Lateral fluoroscopic projection with a percutaneous needle positioned in the L4-5 disc demonstrates opacification of a ventral paraspinous discal cyst (dot) which clearly communicates with the L4-5 disc space (arrowheads) through a narrow neck (arrow). (b) Axial unenhanced pre-procedural CT reveals a hypoattenuating (21 HU) cystic lesion in the left anterior paravertebral space (arrowheads). (c) Parasagittal post-discography CT shows opacification of the discal cyst (dot) which clearly communicates (arrow) with the opacified L4-5 intervertebral disc. (d) Axial post-discography CT shows opacification of the cystic lesion with contrast (arowheads). Approximately 3 mL of serosanguinous fluid was aspirated from the cyst.

All discal cysts occurred at a single level per patient, most commonly at L5-S1. Seven discal cysts were left-sided (78%), and seven were paracentral in location (78%). On MRI, discal cysts were T1 hypointense or isointense and T2 hyperintense (Fig. 1). One case demonstrated a fluid-fluid level on T1W and T2W sequences (Fig. 2). Out of six cysts characterized on gadolinium-enhanced MRI, three (50%) demonstrated rim-enhancement and three were non-enhancing. One patient underwent diagnostic CT and fluoroscopic-guided discography for a ventral paraspinous discal cyst, followed by therapeutic aspiration. This discal cyst measured 21 Hounsfield units (HU) on pre-procedural CT and opacified with contrast during discography confirming communication with the intervertebral disc (Fig. 3).

**Fig. 4:**
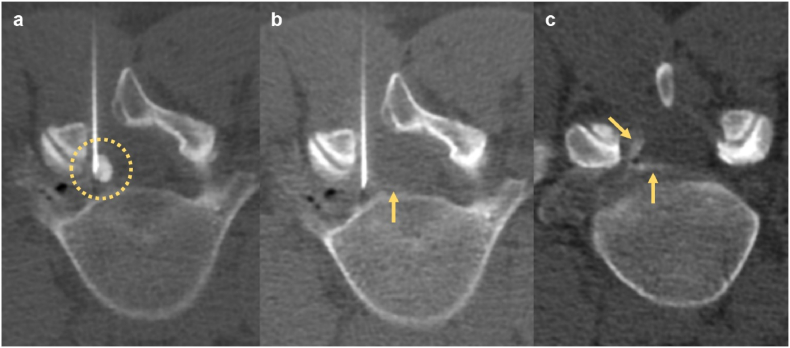
CT-guided discal cyst aspiration, fenestration and rupture in a 30 year old female with a post-operative left L4-5 discal cyst following prior same-level discectomy. (a) Axial intra-procedural CT demonstrates percutaneous needle placement within a left paracentral discal cyst, opacified with contrast (open circle). (b) Axial intra-procedural CT following initial cyst aspiration confirms absence of residual contrast in the cyst. Note the absence of contrast in the ventral epidural space (arrow). (c) Axial post-procedure CT following fenestration and rupture of the discal cyst and epidural steroid injection demonstrates restoration of normal epidural contours, opacified with contrast (arrows). This confirms complete resolution of the discal cyst.

Six patients underwent initial management with image-guided procedures (Table 1, Supplementary Table 1). Four patients were treated with initial CT-guided epidural steroid injections (ESI), three of whom underwent cyst aspiration and fenestration (Fig. 4). One case was managed with initial fluoroscopy-guided transforaminal ESI (TFESI); and one with fluoroscopic discography and aspiration. One case was managed with primary surgery including cyst excision with ipsilateral hemilaminectomy, facetectomy and foraminotomy. One patient with back pain was managed conservatively and had resolution of pain at long-term (9 years) follow-up. One patient with an incidental asymptomatic discal cyst was managed conservatively.

Immediate post-procedure pain scores and short-term follow-up outcomes were variably reported following primary image-guided steroid injection, aspiration or surgery (Supplementary Table 1). Amongst 4 patients undergoing CT-guided procedures, one patient who was managed with two-level TFESI had a repeat CT-guided TFESI with resolution of leg pain at 15 months follow-up (Supplementary Table 2). One patient with post-microdiscectomy discal cyst had short-term relief with CT-guided ESI on two occasions but was definitively managed with revision microdiscectomy with resolution of pain at 9-month follow-up. One patient with lumbar neurogenic claudication had short-term relief with CT-guided ESI and SNRB but required surgery with bilateral laminoforaminotomies and medial facetectomies.

## 4 Discussion

In our cohort of nine patients (4 female), with mean age 49.1 years, five patients presented with back pain and radiculopathy, two with only back pain, one with only radiculopathy, and one asymptomatic. Most discal cysts occurred at L5-S1, were left-sided, paracentral, with a T2 hypointense rim and variable enhancement. One patient underwent primary cyst resection. Amongst 6 patients who underwent primary image-guided procedures, two had sustained pain relief, three proceeded to surgery, and two were lost to follow-up.

Discal cysts are an uncommon disc disease first described in the English literature in 1999 [1] with current experience limited to case reports and case series. The majority of cases series and reports have originated from Asian centers including Korea [[2], [3], [4], [5], [6], [7], [8]], Japan [9,10], and China [11].

Discal cysts have been described in a younger patient cohort than symptomatic disc herniations, with a reported mean age of 33.5 years [12]. Some 90% of discal cysts are described in male patients, with a predilection for Asian patients [13]. By contrast, our cohort were older (mean 49.1 years) and with a less striking male predominance. Patients with discal cysts in our cohort typically presented with back pain and unilateral single-level radiculopathy, mimicking symptomatic disc herniation [10]. In existing literature, motor deficits and hypoesthesia are reported in approximately 40% and 30% of patients respectively [12]. In our cohort, there was a lower incidence (22%) of neurologic deficits on physical examination, with one asymptomatic case.

Discal cysts are most commonly reported as intraspinal, extradural cysts confined to the ipsilateral lateral recess [2] and most frequently described at L4-L5 [12]. Communication with the adjacent disc is a defining feature on fluoroscopic or CT-guided discography [1,2]. However invasive discography has largely been replaced by MRI [14], now preferred for noninvasive diagnosis [13]. MRI criteria for diagnosis include attachment to a lumbar disc, separation from adjacent facet joints, presence of a capsule, and absence of disc material [2]. By contrast, synovial cysts arise from and communicate with facet joints, disc extrusions contain disc material, and perineural sleeve cysts are meningeal diverticula which communicate with and contain CSF.

The fibrotic capsule of the discal cyst is seen on imaging as a T2 hypointense rim [2] as described in our cohort. This fibrotic capsule accounts for characteristic rim enhancement [13] with non-enhancement of the cyst contents. Calcification has been described in the wall of facet synovial cysts [15] but not in discal cysts. Cysts contents may be variable, usually described as T2 hyperintense and with variable T1 hypointensity depending on presence or absence of hemorrhage [13]. Erosion or remodeling of the adjacent vertebral body can be a useful clue to diagnosis [1]. Communication with the disc via a neck or channel was only seen in 3 of our 8 cases on MRI (38%) but is not required for confident MRI diagnosis [2] as a small neck may be obscured by partial volume artifacts at conventional slice thicknesses and intersection gaps. Our case series spans the gamut of imaging findings, including rare ventral paraspinal discal cysts.

Formation of discal cysts may result from damage to the intervertebral disc annulus fibrosis, focal degeneration and cystic softening of collagenous disc tissue and formation of a reactive pseudomembrane [1]. At CT-guided aspiration, discal cyst walls have been described as firm and filled with serous or bloody contents [6]. At histologic examination, the cyst wall is comprised of fibrous connective tissue with myxoid degeneration, and the central fluid component may be hemorrhagic [2]. Absence of a synovial lining or of disc material histologically help to distinguish these from synovial cysts and disc extrusions respectively [16].

Optimal management of discal cysts remains uncertain with experience limited to case reports and series. Spontaneous partial regression [17] or complete resolution [18] of discal cysts has been reported including after steroid administration. CT-guided percutaneous aspiration has been described as effective in the management of lumbar discal cysts and may result in complete regression [6,19,20]. Much like facet synovial cysts, there is a risk of recurrence following non-surgical management. In one case series of 8 patients who underwent CT-guided aspiration, one patient had a recurrent discal cyst at 3-month follow-up [6]. Of three patients in our cohort who underwent cyst aspiration, one had a recurrent cyst, one did not recur, and one was lost to follow-up. Calcification has been described in the wall of facet synovial cysts [15] and may render them more difficult to aspirate. Wall calcification has not been described in discal cysts to our knowledge.

Surgical management with cystectomy alone or microdiscectomy with cystectomy have both been reported [4]. YAG-laser decompression has also been described as a strategy for management [5]. In one literature review [12], three cases (5.3%) showed spontaneous regression of the cyst after conservative treatment, five cases (9%) showed failure after conservative treatment and referred to surgery, and two cases showed recurrence of the cyst (3.5%) after CT-guided aspiration and microsurgical resection. In our series, patients managed with CT-guided steroid injection have benefited from short-term symptomatic relief prior to surgical management, and there were no recurrent discal cysts following surgical resection (Supplementary Table 2).

Our study is limited due to the retrospective nature of the case series with a relatively small number of cases. There is a small possibility that our search strategy excluded cases in which the diagnosis was not considered. In addition, it is challenging to draw conclusions from a cohort with variable duration of clinical and imaging follow-up. Further prospective research is required to determine optimal management of this condition.

## 5 Conclusions

Discal cysts usually arise from the lower lumbar discs with characteristic imaging findings. Our retrospective cohort includes a wider age group with more heterogeneous clinical features than that described in the literature. CT or fluoroscopy-guided steroid injection provided short-term symptomatic relief with several cases managed definitively with cystectomy, microdiscectomy and/or posterior decompressive surgery.

## Presentation

This work was presented in abstract form at the 26th Annual Symposium of the American Society of Spine Radiology in Charleston, SC, USA on 9 February, 2023.

## Funding

This research did not receive any specific grant from funding agencies in the public, commercial, or not-for-profit sectors.

## Declaration of competing interest

The authors declare that they have no known competing financial interests or personal relationships that could have appeared to influence the work reported in this paper.
