# Supplementary material for: Discal cysts and pseudocysts: Single center experience
Source: Interv Pain Med. 2023 Sep 9;2(3):100278. doi: 10.1016/j.inpm.2023.100278 (PMC11372899; doi:10.1016/j.inpm.2023.100278)
Supplement: Multimedia component 1 [file mmc1.docx]

| **#** | **Prior Surgery** | **Treatment 1** | **Injectate (mL/site)** | **Procedure Findings** | **Pre-Proc Pain (/10)** | **Post- Proc  Pain  (/10)** | **1^st^  F/U (days)** | **F/U Pain (/10)** | **F/U  Comments** | **Imaging  F/U (days)** | **Imaging Outcome** |
| --- | --- | --- | --- | --- | --- | --- | --- | --- | --- | --- | --- |
| 1 | Left L4-L5  microdiscectomy | CT-guided  left L4 and L5 TFESI left L4-5 cyst aspiration + fenestration | Celestone^A^ 2mL | 2mL aspirate serous | 9 | 0 | 20 | 4 | - | 16 | recurrent cyst |
| 2 | 0 | Left L4-5 hemilaminectomy facetectomy, foraminotomy cyst excision with microdissection | NA | extradural cyst  ventral to nerve root serous contents | 6 | NR | 43 | 0 | - | 158 | no residual |
| 3 | Left L5-S1  microdiscectomy | CT-guided left L5-S1 cyst aspiration + fenestration left L5 and S1 TFESI | Celestone**^B^** 1.5mL | 1.5mL aspirate  serosanguinous | 4 | NR | 7 | 4 | - | - | - |
| 4 | 0 | Fluoroscopy-guided left L5 and S1 SNRB | 1 | NR | NR | NR | NR | NR | - | - | - |
| 5 | 0 | CT-guided  bilateral L2-L3 SNRB bilateral L4-5 ILESI | Celestone^A^ 1.5 - 2.0mL | NA | 5 | "improved" | 14 | 5 | 2 days  relief | - | - |
| 6 | 0 | Conservative | NA | NA | 0 | NA |  |  | - | - | - |
| 7 | 0 | Conservative | NA | NA | 6 | NA | 3899 | 0 | - | - | - |
| 8 | L4-5  discectomy | Fluoroscopy-guided  L4-5 discography + aspiration |  | 3mL aspirate serosanguinous | NR | NR | 535 | NR | ongoing back/leg pain | 1165 | no residual |
| 9 | 0 | CT-guided  right L5 and S1 SNRB | Kenalog^C^  1.5mL | NA | 4 | 0 | 31 | 2 | 4 weeks relief | - | - |

**Supplementary Table 1: Short-term outcomes following initial management.** F/U = follow-up; SNRB = selective nerve root block; TFESI = transforaminal epidural steroid injection; ILESI = interlaminar epidural steroid injection; A = 1:1 Celestone (betamethasone) 6 mg/mL + 0.75% bupivacaine; B = 1:1 Celestone (betamethasone) 6 mg/mL + 0.50% bupivacaine; C = 1:2 Kenalog (triamcinolone) 40 mg/mL + 0.75% bupivacaine; NA = not applicable; NR = not reported.

| **#** | **Prior Surgery** | **Treatment 1** | **Pre- Proc**  **Pain  (/10)** | **Treatment 2** | **Definitive**  **Treatment** | **Last F/U**  **Days (M/Y)** | **Last Pain**  **Score (/10)** | **Last F/U  Outcome** |
| --- | --- | --- | --- | --- | --- | --- | --- | --- |
| 1 | Left L4-L5  microdiscectomy | CT-guided  left L4 and L5 TFESI left L4-5 cyst aspiration + fenestration | 9 | CT-guided TFESI left L4-5 & left L5-S1 | Revision L4-5 microdiscectomy | 272  (≈9 months) | 1 | near-resolution of pain |
| 2 | 0 | Left L4-5 hemilaminectomy facetectomy, foraminotomy cyst excision with microdissection | 6 | - | 1° surgery | 365  (=1 year) | 0 | resolved pain |
| 3 | Left L5-S1  microdiscectomy | CT-guided left L5-S1 cyst aspiration + fenestration left L5 and S1 TFESI | 4 | - | 1° image-guided aspiration/TFESI | 7  (=1 week) | 4 | lost to follow-up |
| 4 | 0 | Fluoroscopy-guided left L5 and S1 SNRB | NR | - | 1° image-guided SNRB | - | - | lost to follow-up |
| 5 | 0 | CT-guided  bilateral L2-L3 SNRB bilateral L4-5 ILESI | 5 | - | L2-3 bilateral  laminotomy  foraminotomy  medial facetectomy | 244  (≈8 months) | NR | resolved hypesthesia,  residual paresthesia |
| 6 | 0 | Conservative | 0 | - | Conservative | 0 | 0 | - |
| 7 | 0 | Conservative | 6 | - | Conservative | 3633  (≈ 10 years) | 0 | resolved pain, residual L5 hypesthesia |
| 8 | L4-5  discectomy | Fluoroscopy-guided  L4-5 discography + aspiration | NR | - | 1° image-guided aspiration | 3331  (≈ 9.1 years) | 2 | chronic back & bilateral leg pain |
| 9 | 0 | CT-guided  right L5 and S1 SNRB | 4 | - | CT-guided  right L5 and S2 SNRB  right L5-S1 ILESI | 466  (≈ 1.3 years) | 0 | resolved leg pain  residual mild back pain |

**Supplementary Table 2: Long-term management and outcomes.** F/U = follow-up; SNRB = selective nerve root block; TFESI = transforaminal epidural steroid injection; ILESI = interlaminar epidural steroid injection; NA = not applicable; NR = not reported.
